# Extent of cancer at the time of diagnosis in older adults: a cohort of a Chilean cancer insurance program (2018–2022)

**DOI:** 10.3332/ecancer.2026.2114

**Published:** 2026-04-29

**Authors:** Sergio Muñoz-Navarro, Christian Caglevic, Jorge Sapunar

**Affiliations:** 1 Cancer Research Department, Arturo López Pérez Oncology Foundation Institute, Rancagua Avenue 878, Santiago 7500000, Chile; 2 CIGES Centre of Excellence, Faculty of Medicine, University of La Frontera, Manuel Montt Avenue 112, Office 310, Temuco 4781176, Chile; 3 Department of Internal Medicine, Faculty of Medicine, University of La Frontera, Manuel Montt Avenue 112, Office 422, Temuco 4781176, Chile

**Keywords:** epidemiology, cancer staging, older adults

## Abstract

**Introduction:**

In Chile, the cancer mortality rate ratio between older adults and individuals under 65 years of age is 12.14. This excess mortality may be attributed to cancer-specific factors or comorbidities commonly observed in older adults.

**Objective:**

To compare the frequency of advanced clinical stages at diagnosis, for the five most epidemiologically significant malignant neoplasms – breast, prostate, lung, colorectal and gastric cancers – between older adults (≥65 years) and individuals between 40 and 64 years of age.

**Methods:**

We conducted a retrospective study of health insurance beneficiaries, identifying new cases of breast, prostate, lung, colorectal and gastric cancers diagnosed between 2018 and 2022. Age-adjusted incidence rates and the proportion of advanced clinical stages (III and IV) were compared between older adults and younger individuals.

**Results:**

The cancer incidence rate ratio between older and younger adults ranged from 1.77 to 6.315 (p < 0.0001). For lung, colorectal and gastric cancers, the majority of patients were diagnosed at advanced clinical stages. Conversely, for breast and prostate cancers, the proportion of advanced stages was 18.07% and 40.37%, respectively. Older adults exhibited a significantly higher risk of advanced-stage diagnosis only for gastric cancer.

**Discussion:**

The incidence of major cancers was markedly higher among individuals aged 65 and older. However, there were no significant differences in the proportion of advanced clinical stages at diagnosis across most cancer types, except for gastric cancer. The elevated cancer-related mortality observed in older adults may be more closely associated with comorbid conditions rather than the stage at diagnosis.

## Introduction

According to United Nations estimates, the proportion of individuals aged 65 years or older has increased from 5.1% in 1963 to 10% in 2023 [1]. This demographic shift is more pronounced in regions with a high Human Development Index (HDI), such as Europe and North America, where the proportion rose from 9% in 1963 to 19.2% in 2023. However, this trend is also evident in regions with medium to low HDIs, such as Latin America,

where the proportion increased from 4.46% in 1963 to 5.83% in 2023 [1]. In Chile, the proportion of older adults rose from 5% in 1960 to 11.4% in 2017, as observed in successive population censuses [2, 3]. Current life expectancy in Chile is 76 years for men and 81 years for women [4]. Alongside the increasing proportion of older adults, the absolute population has also grown, with global estimates indicating 833 million older adults in 2024, projected to rise to 1.578 billion by 2050 [1]. For Chile, the number of older adults is estimated at 2.8 million in 2024 and expected to reach 5.4 million by 2050 [1].

The public health implications of this growing older population include increased utilisation of healthcare services and associated costs, attributable to a higher prevalence of chronic diseases, including cancer, multi-morbidity and age-related disabilities [5, 6]. Furthermore, ageing is now recognised as one of the primary, if not the most significant, risk factors for cancer development [7].

The projected cancer incidence for 2022 by the International Agency for Research on Cancer (GLOBOCAN) ranged from 409 cases per 100,000 inhabitants in Oceania (a region with a very high HDI) to 132.3 cases per 100,000 in Africa (a region with a low HDI) [8]. When comparing cancer incidence in older adults to the rest of the adult population using the incidence rate ratio (IRR), the IRR was 6.84 in Oceania and 6.01 in Africa [8]. This indicates that, regardless of HDI, older adults have a significantly higher cancer incidence than the rest of the adult population. In Latin America, the estimated cancer incidence by GLOBOCAN for 2022 was 186 cases per 100,000 inhabitants, with an IRR of 7.46 between older adults and the general adult population [8]. In Chile, the estimated cancer incidence was 188.7 cases per 100,000 inhabitants, with an IRR of 9.5 between older adults and the rest of the adult population [8].

In addition to the higher frequency of cancer among older adults, various epidemiological factors contribute to increased cancer lethality. The cancer mortality rate ratio (MRR) between older adults and the general adult population was 13.2 in Oceania and 7.86 in Africa. In Latin America, the estimated mortality rate for 2022 was 85.5 deaths per 100,000 inhabitants, with an MRR of 10.98 between older adults and the rest of the adult population [8]. In Chile, the cancer mortality rate in 2018 reached 100.1 deaths per 100,000 inhabitants, making cancer the leading cause of death [9]. The MRR for cancer between older adults and the rest of the adult population in Chile, based on GLOBOCAN 2022 data, was 12.14.

Comorbidities are strongly associated with increased cancer mortality in older adults. According to Chile’s 2016–2017 National Health Survey, 65.5% of older adults had increased cardiovascular risk, 74% had hypertension and approximately 30% had diabetes mellitus [10].

Most countries with medium or low HDIs, including Latin America, lack adequate population-based tumour registries [11, 12], which limits the validity of cancer incidence estimates provided by GLOBOCAN because they are usually projections of partial data from the past time. Furthermore, attributing a cause of death to cancer in older adults with multiple comorbidities can be challenging.

Given the absence of a national tumour registry in Chile and other countries in Latin America, this study aimed to compare the incidence rates of the five most epidemiologically significant malignant neoplasms in the country between older adults and adults between 40 and 64 years of age treated for their neoplasms at a single institution. Additionally, the study evaluated the frequency of advanced clinical stages at diagnosis across these two age groups to assess the role of late diagnosis in the higher cancer-related mortality observed in older adults.

## Patients and methods

This cross-sectional study focused on all health insurance beneficiaries aged 40 years or older enrolled in the ‘Oncology Agreement’ of the Oncology Foundation Institute (FALP) as of December 31 between 2018 and 2022. Using the FALP Tumour Registry, we identified all new cases of breast, prostate, lung, colorectal and gastric cancers diagnosed in individuals aged 40 years or older during this period by activating cancer insurance. The type of malignancy, age, sex and clinical stage at diagnosis and date of diagnosis were recorded.

A case was considered incidental if it was histologically confirmed. Due to the nature of the insurance coverage, there were no incidental cases among newly enrolled individuals. When two or more primary cancers were detected in a single person, they were considered independent cases. For metachronous diagnoses during the follow-up period, only the first diagnosis was considered.

Cancer incidence rates were calculated by cancer type and year of diagnosis for the entire study cohort and subsequently stratified by age into two groups: older adults aged 65 years or older and individuals aged 40–64 years. In both groups, incidence rates were adjusted for age using the direct method. For malignancies exclusive or predominant in one sex, such as prostate and breast cancers, incidence rates were calculated for the respective subgroups.

Since anonymised institutional registry data were used, informed consent was waived. The study protocol was approved by the Ethics Committee (Clinical Research Protocol No 2024-429-EPI-SIN-INT).

We described the age-adjusted incidence of breast, prostate, lung, colorectal and gastric cancers by year, sex (where relevant) and age group as described above. To establish the differences between the population of Chile (Census 2018) [3] and our cohort, we compared the distribution by sex and age stratum using goodness of fit. The frequency of clinical stages was reported by year, sex and age group based on the AJCC/TNM 8th Edition criteria for each type of cancer [13]. Annualised incidence rates for different types of cancers were compared between older adults and younger individuals using IRRs through Poisson regression models. We check for over dispersion and, if present, readjust it with a negative binomial model. Similarly, the frequency of advanced clinical stages (III and IV) was compared between age groups using the prevalence ratio (PR) in a multivariate model. Cases with unreported staging were excluded from analysis. The significance level was 0.05.

Data management and hypothesis testing were conducted using STATA 18.0 (StataCorp. 2023. Stata Statistical Software: Release 18. College Station, TX: StataCorp LLC).

## Results

Table 1 presents the health insurance beneficiaries aged 40 years or older enrolled in the FALP Oncology Agreement between 2018 and 2022, along with the number of new cases of breast, prostate, lung, colorectal and gastric cancers, stratified by sex where relevant. When comparing the cohort with the population of the 2018 census over 40 years of age, significant differences are observed in the distribution by age and sex.

**Figure 1.**
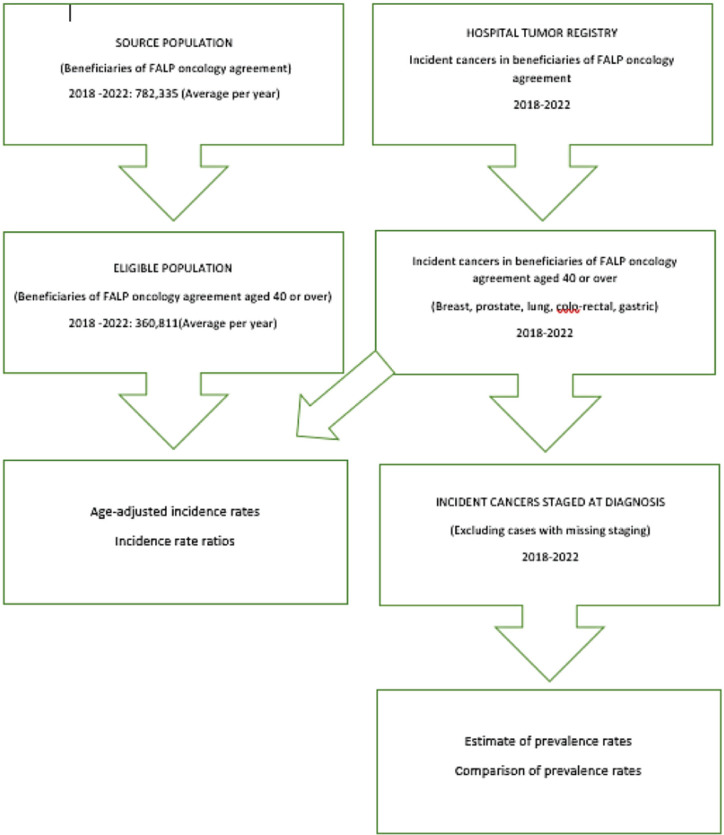
Study flow diagram.

Figure 1 shows the flow of the study.

**Figure 2.**
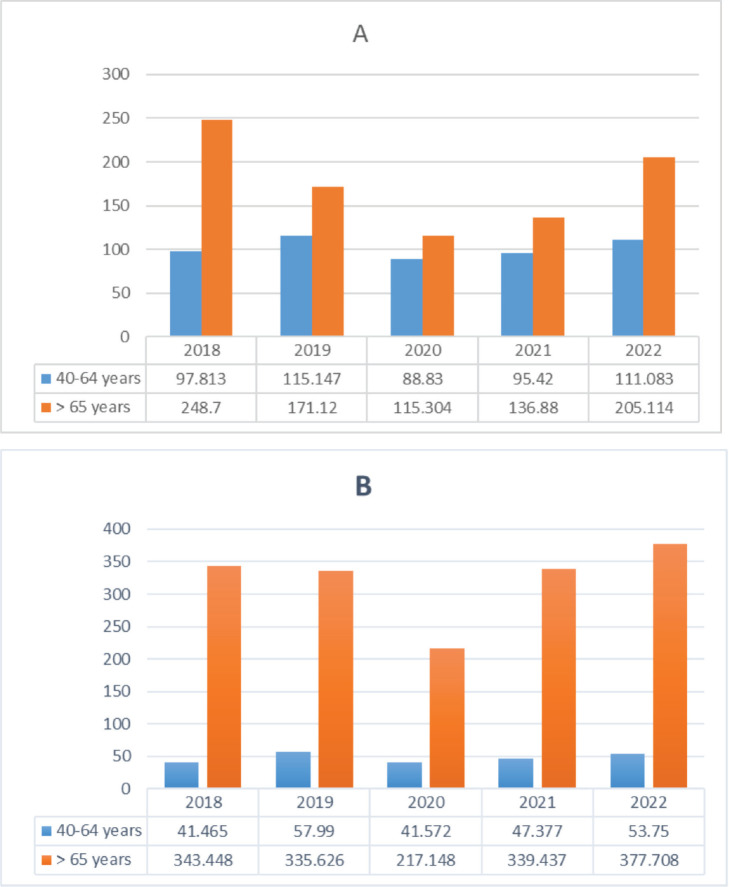
Age-adjusted incidence of breast cancer (a) in older women and in the rest of the female cohort over 40 years of age in the FALP oncological agreement between 2018 and 2022. Age-adjusted incidence of prostate cancer (b) in older men and in the rest of the male cohort over 40 years of age with FALP Oncology Agreement between 2018 and 2022 (cases per 100,000).

Figure 2a,b shows age-adjusted incidence rates for breast and prostate cancers from 2018 to 2022 in both older adults and younger individuals. After adjusting for year of diagnosis using Poisson regression, we observed over dispersion, so the IRRs were estimated using a negative binomial model. IRR of breast cancer is 1.73 higher in older adults compared to the rest of the population (95%CI: 1.41–2.12). For prostate cancer, the IRR is 5.11 (95% CI: 2.63–9.92).

**Figure 3.**
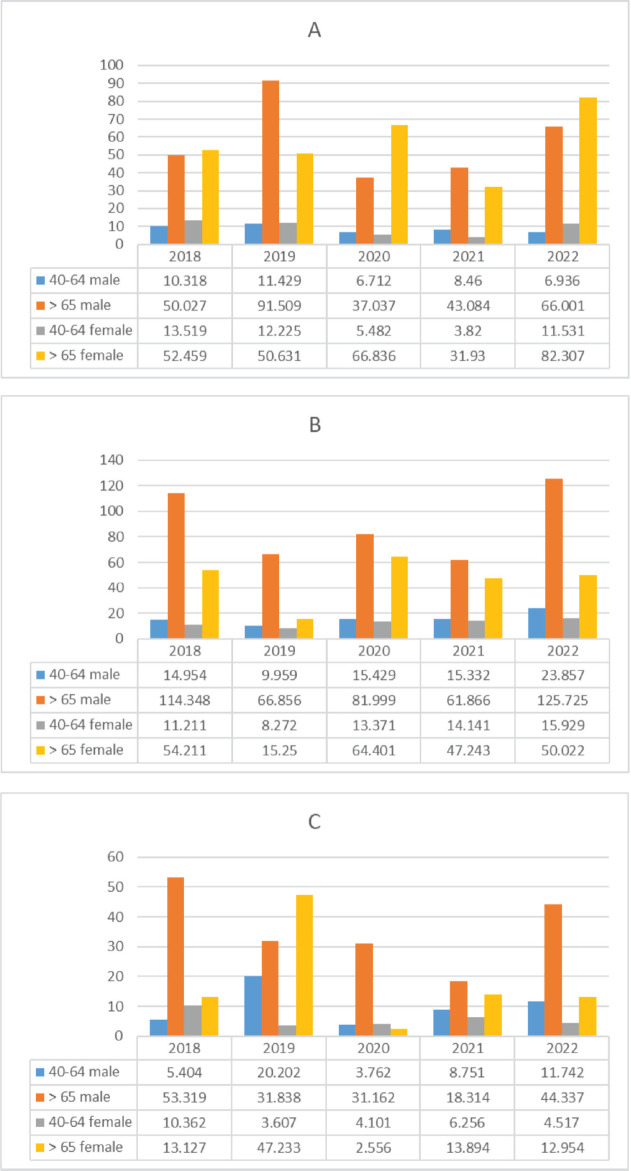
Age-adjusted incidence of lung (a), colorectal (b) and gastric (c) cancer in older adults and in the rest of the population over 18 years of age with FALP Oncological Agreement, by sex, between 2018 and 2022 (cases per 100,000).

Figure 3 illustrates the age-adjusted incidence rates for lung, colorectal and gastric cancers by sex from 2018 to 2022 in older adults and younger individuals. Adjusting for sex and year using Poisson regression, we observed over dispersion, so the IRRs were estimated using a negative binomial model. The IRR was 4.47 for lung cancer (95% CI: 2.68–7.47), 2.78 for colorectal cancer (95% CI: 1.99–3.87) and 2.59 for gastric cancer (95% CI: 1.62–4.16). Male sex was associated with an IRR of 1.68 for colorectal cancer (95% CI: 1.22–2.31) and 2.07 for gastric cancer (95% CI: 1.31–3.26). A decrease in cancer incidence, particularly among older adults, was observed in 2020 during the COVID-19 pandemic; however, this reduction was only statistically significant for breast and prostate cancer.

Table 2 reports the frequency of advanced clinical stages (III and IV) by AJCC/TNM 8th Edition criteria at diagnosis, stratified by year and sex. Most lung, colorectal and gastric cancer cases were diagnosed at advanced clinical stages. In contrast, only 18.07% of breast cancer cases and 40.37% of prostate cancer cases were diagnosed at advanced stages. The proportion of unstaging cases was 7% in lung cancer, 10% in breast cancer, 13% in colorectal cancer, 19% in prostate cancer and 22% in gastric cancer. There were no differences in the proportion of unstaging cases between older adults and the rest of the cohort.

Table 3 presents the PR of advanced clinical stages (III and IV) for older adults compared to younger individuals for prostate, breast, lung, colorectal and gastric cancers. The PR was significantly higher for gastric, with a trend toward lower risk for breast cancer, though this was not statistically significant.

## Discussion

GLOBOCAN estimated the cancer age-adjusted incidence rates in Chilean adults aged 40 and over for 2022 as follows: 38.9 cases per 100,000 inhabitants for lung cancer, 61.7 cases per 100,000 for colorectal cancer and 43.5 cases per 100,000 for gastric cancer. Based on these rates, the IRR between older adults and the general adult population was 7.67 for lung cancer, 4.86 for colorectal cancer and 6.63 for gastric cancer [8]. In our study, conducted with beneficiaries of the FALP Oncology Agreement in Chile, the age-adjusted incidence rates in 2022 were 32.14 cases per 100,000 beneficiaries for lung cancer, 19.02 cases per 100,000 for colorectal cancer and 14.45 cases per 100,000 for gastric cancer. The IRR for older adults compared to younger adults was 7.82 for lung cancer, 4.1 for colorectal cancer and 3.21 for gastric cancer. Although both sources demonstrated substantially higher cancer incidence rates in older adults, the absolute rates differed significantly between datasets.

Regarding breast cancer, GLOBOCAN estimated an age-adjusted incidence of 103 cases per 100,000 women aged 40 and over in Chile for 2022, with an IRR of 1.69 between older and younger adult women [8]. In our study, the incidence rate was 205.114 cases per 100,000

women, with an IRR of 1.85. For prostate cancer, GLOBOCAN estimated an age-adjusted incidence rate of 192.4 cases per 100,000 men aged 40 and over in Chile for 2022, with an IRR of 9.19 between older and younger men [8]. In our study, the prostate cancer incidence rate was 130.454 cases per 100,000 men, with an IRR of 7.09. Despite differences in absolute rates, our findings align with GLOBOCAN’s estimates regarding the disproportionately higher incidence of breast and prostate cancers in older adults.

GLOBOCAN's cancer incidence estimates for Chile are based on data reported by seven sentinel centers across Chile. These data have not been updated since 2018 and include new cases from only 20% of the Chilean population, omitting the most populated geographical area of the country. Although the differences observed in rates could be attributed to the fact that they are different populations, it must be considered that the GLOBOCAN rates are projections of data obtained from a sample of the population that it is intended to represent, in a past time. The possibility of underreporting in our study is low, given the closed nature of the care provided to insurance beneficiaries.

In terms of mortality, GLOBOCAN estimated mortality rates in Chile for 2022 as follows: 16.4 deaths per 100,000 inhabitants for lung cancer, 13.4 deaths per 100,000 for colorectal cancer and 16 deaths per 100,000 for gastric cancer. The MRRbetween older adults and the general adult population was 18.6 for lung cancer, 15.64 for colorectal cancer and 16.2 for gastric cancer [8]. Since these rates reflect overall mortality, it is expected that older adults with cancer have significantly higher figures, although this does not necessarily indicate that cancer was the direct cause of death.

One factor that could be associated with the higher cancer mortality observed in older adults is diagnosis at more advanced clinical stages compared to younger adults, which in turn could be associated with less access to health care. Our results only showed a significantly higher proportion of advanced-stage (III and IV) diagnoses among older adults with gastric cancer compared to younger adults. A Korean study comparing clinic-pathological variables between older adults and younger individuals (<36 years) with gastric cancer found no significant differences in clinical staging [14]. This discrepancy may be explained by the comparison groups, as we evaluated older adults against the general adult population, while the Korean study compared older adults with a much younger subgroup. Another Korean report compared patients aged 75 years and older to those aged 65–74 years and found significantly higher proportions of stage III and IV gastric cancers among the older group [15]. These findings suggest a possible association between older age and more advanced-stage gastric cancer at diagnosis.

For breast cancer, evidence remains controversial. A comparison of two cohorts of older Italian women with breast cancer revealed a trend toward less advanced clinical stages over time in this age group [16]. However, a Dutch study reported that women aged 65 years and older were diagnosed at more advanced stages, underwent fewer oncological surgeries, received less adjuvant chemotherapy, were more likely to receive hormonal therapies, and exhibited higher overall mortality compared to younger patients. Interestingly, in the subgroup of women aged 75 years and older, cancer-associated mortality was lower than 50% [17].

Colorectal cancer exhibits biological differences between younger and older patients, with a tendency for greater aggressiveness and more advanced stages in younger individuals. However, population-based studies have confirmed similar survival outcomes across both groups for stage I, II and III disease. Conversely, in metastatic disease, progression-free survival tends to be worse in younger patients compared to older adults [18].

Lung cancer is significantly more prevalent among older adults than younger individuals. Nevertheless, reports from the United States suggest that lung cancer can be effectively diagnosed and treated with curative intent, even in advanced age [19].

The primary limitation of our study is that the results should be extrapolated to the Chilean population with caution, as there are demographic and socioeconomic differences between the general population and the FALP Oncology Agreement beneficiaries. However, given both global and local trends of an ageing population and the high IRR observed for major malignancies in older adults, we anticipate a progressive increase in healthcare demand for this demographic. Consistent with other studies [19–23], our findings show that lung, colorectal and gastric cancers are predominantly diagnosed at advanced clinical stages, particularly in older adults. Conversely, breast cancer among women aged 65 years and older appears to present with a more favourable clinical profile.

Further research is needed to determine whether the higher cancer-related mortality in older adults is primarily associated to the biological behaviour of malignancies or the comorbidities characteristic of this age group. Along with the above, other factors that could be associated with the excess mortality from cancer in older adults are the decision not to treat due to age, the lack of evidence due to not including them in clinical trials and the recommendations of clinical practice guidelines. Finally, access to health care in older adults is usually more limited [24]. The differences observed between our study and international experiences underscore the need to strengthen early cancer detection strategies, particularly for older adults, enhance interdisciplinary discussions when defining oncological treatments, and, most importantly, develop onco-geriatric programs and units to facilitate more effective and less detrimental therapeutic decisions, accounting for the unique needs of older adults compared to younger cancer patients.

## Glossary

**Human Develop Index**: Statistical composite index of life expectancy, education (mean years of schooling completed and expected years of schooling upon entering the education system) and per capita income indicators.

**Incidence rate ratio:** Incidence rate in the study group divided by the incidence rate in the control group.

**Mortality rate ratio**: Mortality rate in the study group divided by the mortality rate in the unexposed group.

**Prevalence ratio**: Ratio of the proportion of a variable in two comparison groups.

**Clinical stage**: Is an estimate of the extent of the cancer based on results of physical exams, imaging tests (x-rays, CT scans and so on), endoscopy exams and any biopsies that are done before treatment starts. For the carcinomas included in the study, we used the 8th Edition of the AJCC/TNM classification system [12].

## Author contributions

Sergio Muñoz Navarro: Data curation, Formal analysis, Methodology, Writing – original draft. Christan Caglevic Medina: Conceptualisation, Formal analysis, Investigation, Writing – original draft. Jorge Sapunar Zenteno: Conceptualisation, Formal analysis, Methodology, Project administration, Writing – original draft, Writing – review and editing.

## Conflicts of interest

Sergio Muñoz: No conflicts to disclose. Christian Caglevic: Investigator: MSD, AZ, Roche, Astellas Pharma, BMS, Athenex, SPaanofi, Abbvie, Amgen, Bayer H P, Biontech SE, Daiichi Sankyo INC, Exelixis, Novartis, F Hoffmann LaRoche, PharmaMar, Zymeworks, Cogent Biosciences, Pfizer, Dizal Pharma, Advisore – Consulting: Gilead Sciences. Jorge Sapunar: No conflicts to disclose.

## Funding

This research did not receive any specific grant from funding agencies in the public, commercial or not-for-profit sectors. No sponsor influenced the design, analysis or reporting of the study.

## Figures and Tables

**Table 1.** Population over 40 years of age in the FALP Oncological Agreement and new cases of breast, prostate, lung, colorectal and gastric carcinomas during the years 2018, 2019, 2020, 2021 and 2022, according to sex when applicable.

Population	2018	2019	2020	2021	2022
Male	116,327	161,748	161,426	172,066	189,398
Female	176,523	195,896	195,854	207,078	227,741
Total	292,850	357,644	357.280	379,144	417,139
New cases of cancer	2018	2019	2020	2021	2022
Breast	203	237	186	205	279
Prostate	132	172	119	171	205
Lung					
Male	24	34	21	24	30
Female	26	34	26	17	39
Total	50	68	47	41	69
Colorectal					
Male	46	31	43	41	80
Female	32	20	47	42	52
Total	78	51	88	83	131
Gastric					
Male	23	34	18	20	35
Female	17	21	7	16	11
Total	40	55	25	36	46

**Table 2.** Proportion of new cases of breast, prostate, lung, colorectal and gastric carcinomas in clinical stage III–IV at the time of diagnosis, in the years 2018, 2019, 2020, 2021 and 2022, according to sex when applicable.

Location	2018 N (%)	2019 N (%)	2020 N (%)	2021 N (%)	2022 N (%)	Period complete
Breast	35 (17.41)	44 (18.64)	33 (17.28)	44 (23.04)	43 (15.25)	199 (18.07)
Prostate	41 (42.27)	45 (40.54)	42 (37.84)	50 (40.00)	65 (41.14)	243 (40.37)
Lung						
Male	17 (80.95)	25 (83.33)	18 (90.00)	19 (82.61)	19 (67.86)	98 (80.33)
Female	21 (80.77)	19 (65.52)	20 (86.96)	13 (76.47)	23 (62.16)	96 (72.73)
Total	38 (80.5)	44 (74.58)	38 (88.77)	32 (80.00)	42 (64.62)	194 (76.38)
Colorectal						
Male	32 (74.42)	15 (55.56)	19 (50.00)	19 (51.35)	49 (63.64)	134 (60.36)
Female	16 (59.26)	13 (68.42)	28 (62.22)	23 (60.53)	28 (65.12)	108 (62.79)
Total	48 (68.57)	28 (60.87)	47 (56.63)	42 (56.00)	77 (64.17)	242 (61.42)
Gastric						
Male	10 (76.92)	14 (53.85)	12 (70.59)	10 (58.82)	23 (85.19)	69 (69.00)
Female	10 (83.33)	6 (37.50)	5 (71.43)	11 (78.57)	8 (80.00)	40 (67.80)
Total	20 (80.00)	20 (47.62)	17 (70.83)	21 (67.74)	31 (83.78)	109 (68.55)

**Table 3.** Prevalence ratio (PR) of having advanced clinical stage (III–IV) at the time of diagnosis as an older adult with breast, prostate, lung, colorectal and gastric carcinomas, considering year and sex.

Cancer	PR	95% CI	Valor p
Prostate	1.21	0.99–1.48	0.062
Breast	0.71	0.51–1.01	0.057
Lung	0.95	0.83–1.09	0.488
Colorectal	0.94	0.80–1.10	0.477
Gastric	1.32	1.13–1.54	<0.0001
